# Strategies for Photoelectrochemical Splitting of Water

**DOI:** 10.3390/ijms27073015

**Published:** 2026-03-26

**Authors:** Brisa Alejandra Ortiz, Martin Trejo-Valdez, Puja Kumari, Carlos Torres-Torres

**Affiliations:** 1 Sección de Estudios de Posgrado e Investigación, Escuela Superior de Ingeniería Mecánica y Eléctrica Unidad Zacatenco, Instituto Politécnico Nacional, México City 07738, Mexico; 2 Escuela Superior de Ingeniería Química e Industrias Extractivas, Instituto Politécnico Nacional, Ciudad de México 07738, Mexico; 3 Department of Physics, New Government Polytechnic, Patna 800013, India

**Keywords:** photoelectrochemistry, solar fuels, water splitting, hydrogen production, oxygen evolution reaction, photoelectrodes

## Abstract

The photoelectrochemical splitting (PEC) of water provides a direct route to converting solar energy into storable chemical fuels. When illuminated, a semiconductor photoelectrode can absorb light and generate electron-hole pairs, which participate in interfacial redox reactions at the semiconductor-electrolyte junction. Therefore, to achieve high-performance PEC, photoelectrodes with optimized optical absorption and charge have been explored. This review analyzes recent fabrication strategies used to design photoelectrodes for the PEC dissociation of water. Physical fabrication techniques, including pulsed laser deposition, magnetron sputtering, and physical vapor deposition, allow for precise control of film thickness, crystallinity, and defect density, critical parameters for efficient charge transport. Typically, in physical methods, reported photocurrent densities span from ~10^−2^ to 10^1^ mAcm^−2^, depending on the semiconductor material, nanostructure design, and interfacial engineering strategies. Chemical synthesis methods, such as hydrothermal growth, successive ion layer adsorption and reaction, and microemulsion techniques, provide greater compositional flexibility and enable controlled doping, surface functionalization, and the formation of nanostructured morphologies. Finally, hybrid fabrication strategies integrate physical and chemical processes within a single synthesis framework to combine structural precision with compositional tuning capabilities. These approaches enable the development of advanced architecture such as heterojunctions, core–shell nanostructures, and catalyst-modified interfaces, which enhance light absorption and optimize interfacial transfer. Furthermore, theoretical and computational tools are here analyzed as complementary approaches that guide the rational design and optimization of photoelectrochemical materials and devices.

## 1. Introduction

The photoelectrochemical splitting (PEC) of water represents a viable way to convert solar energy into storable chemical fuels, with hydrogen as the main target [1,2,3]. The design and synthesis of semiconductor materials capable of efficiently performing light absorption can effectively generate and separate charge carriers in order to maintain sustained interfacial redox activity under prolonged operating conditions [4,5,6,7]. Numerous design and manufacturing approaches, each with unique benefits and drawbacks, have been investigated because of ongoing developments in photoelectrochemical materials [8,9]. In this context, physical, chemical, hybrid, and theoretical approaches can be viewed as four complementary methodological paradigms related to current research on PEC systems [10,11,12].

Physical manufacturing techniques rely on mechanical, electromagnetic, or plasma-based energy transfer to deposit and structure thin films and nanostructures [13,14]. These approaches provide fine control over key material parameters such as density, crystallinity, and composition [15,16]. Methods, including pulsed laser deposition (PLD), magnetron sputtering, physical vapor deposition (PVD), and anodization, are therefore widely applied in the preparation of photoelectrodes, as they enable high material purity, well-defined stoichiometry, and relatively low defect densities [17,18,19,20,21].

These characteristics, namely high material purity and reduced defect densities, are critical for optimizing key charge-transport parameters, including charge-carrier separation, mobility, and recombination dynamics [22,23]. In addition, many physical manufacturing techniques are compatible with large-area and high-throughput manufacturing approaches, such as roll-to-roll processing and laser-based patterning, which makes them well-suited for the scalable implementation of photoelectrochemical devices [24]. Given the intrinsic characteristics of individual manufacturing routes, such as the limited compositional flexibility of purely physical techniques and the reduced atomic-level control often associated with chemical methods, hybrid fabrication strategies have gained increasing attention [25,26]. These approaches integrate physical and chemical processes within a single synthesis pathway, allowing complementary benefits to be combined [27,28].

For example, coupling hydrothermal growth with successive ion layer adsorption and reaction (SILAR) based sensitization, or employing photodeposition on prestructured substrates, enables the fabrication of complex and controlled photoelectrode architectures [29,30]. Representative configurations incorporated core–shell nanostructures [31], cascade heterojunctions, and cocatalyst-modified surfaces [32,33]. These designs enable multiscale control over morphology, charge-transport pathways, and interfacial band alignment [34,35]. This level of control is associated with higher quantum efficiency and greater photostability [36,37], primarily through reduced recombination losses and more stable surface reaction pathways [38,39]. Furthermore, hybrid strategies enable the integration of additional functionalities, such as internal electric fields [40], hierarchical porosity [41], and plasmonic components, which directly influence light capture and charge utilization, thereby improving the performance of photoelectrochemical systems for hydrogen production [42,43].

Theoretical and computational methods have emerged as essential instruments for the logical design and optimization of photoelectrochemical materials and devices in tandem with experimental developments [44,45]. Electronic structure [46], defect energies [47], dopant incorporation [48], and surface stability of materials are all frequently examined using first-principles calculations [49]. Density functional theory (DFT) is a quantum mechanical computational method used to investigate the electronic structure of atoms, molecules, and solid-state materials; instead of solving the many-body wavefunction of electrons directly. DFT describes the system in terms of its electron density, which significantly reduces computational complexity while still providing accurate predictions of electronic properties, band structures, adsorption energies, and catalytic reaction pathways, which calculations in the literature are commonly performed using widely adopted computational packages depending on the specific study [50,51]. Furthermore, thermal effects, crystal lattice distortions, and structural stability under operating conditions are all revealed by molecular dynamics (MD) simulations [52]. Optimizing geometry and light management techniques at the device level through optical modeling and ray tracing simulations creates a clear connection between material characteristics and overall photoelectrochemical performance [53,54]. Similarly, gas evolution, electrolyte flow [55], and mass transport phenomena in photoelectrochemical [56] reactors are increasingly being analyzed using computational fluid dynamics (CFD) and hydrodynamic modeling [57]. More recently, data-driven approaches [58], including machine-learning-assisted screening and material optimization [59], have been applied to accelerate the identification of promising photoelectrodes, thereby reducing reliance on purely empirical, trial-and-error strategies [60,61].

With regard to these considerations, this work focuses specifically on the fabrication techniques used to design photoelectrodes for PEC systems [62,63]; although numerous reviews have already addressed the photoelectrochemical dissociation of water from perspectives such as catalyst development [64], semiconductor materials [12], reactor engineering [65], and device performance [66,67]. This review focuses on how various fabrication fields (physical, chemical, hybrid, and computational) contribute to the structural and functional optimization of PEC photoelectrodes [68], rather than on examining specific materials or catalytic mechanisms in isolation [69,70].

The analysis in this review aims to make clear how fabrication approaches can guide the logical design of next-generation PEC systems for the efficient production of solar hydrogen, classifying the current literature according to fabrication strategies and comparing their advantages, disadvantages, and integration potential. To support this study, a comprehensive literature review was conducted, including studies published within the last ten years.

The search focused on peer-reviewed articles and review papers on fabrication techniques, heterostructure engineering, nanostructured photoelectrodes, and performance optimization in photoelectrochemical water dissociation systems. Major databases, such as MDPI, Elsevier (ScienceDirect), ACS publications, Royal Society of Chemistry and ResearchGate. In order to identify representative developments and emerging trends in this field, reference studies were selected based on their fabrication parameters and their photoelectrochemical performance.

The main systems included in this review are presented in Table 1 to provide a clearer overview of the materials frequently studied for the photoelectrochemical water splitting. This table lists representative photoelectrode materials.

## 2. Fabrication Strategies for Photoelectrochemical Water Splitting Systems

This section describes the methodological framework adopted to analyze manufacturing strategies in photoelectrochemical systems. Physical, chemical, hybrid, and computational approaches were evaluated through a critical review of recent literature, with emphasis on experimental procedures, the most relevant processing parameters, and reported performance metrics related to photoelectrode functionality and device operation.

### 2.1. Physical Fabrication Methods

Physical fabrication techniques are based on mechanical, electromagnetic or plasma-mediated energy transfer processes that allow the deposition or structuring of photoactive materials with a high degree of control over composition and microstructure. Representative methods include PLD [71], magnetron sputtering [72], and anodization [73], which are widely used in the fabrication of photoelectrodes for photoelectrochemical applications [74,75].

Each technique was evaluated by considering its key operating parameters, such as laser fluence, working pressure, substrate temperature, and deposition time, and their impact on film morphology and crystallinity [76,77]. In addition, reported photoelectrochemical performance metrics, including photocurrent density, incident photon-to-current efficiency (IPCE), and hydrogen evolution rate, were used to evaluate the functional relevance of the resulting photoelectrodes [78,79].

Representative studies employing physical manufacturing methods for photoelectrochemical electrodes can be related to nanoscale materials, perovskites, inorganic and organic semiconductors, plasmonic nanoparticles and nanohybrids [80,81,82,83,84,85,86]. The impact of these elements on the manufacturing strategies provides manufacturing parameters and performance metrics for photoelectrodes. The physical methods that can be analyzed in their performance correspond to magnetron sputtering, pulsed laser epitaxy, 3D printing, terminal sputtering, PLD, electrochemical reduction and micro contact patterning.

Key results demonstrate progress in improved charge separation, efficient charge separation and carrier mobility, enhanced light trapping, enhanced oxygen vacancies, higher activity, and doubled photocurrent. The responsible parameters in this performance are layer thickness, band matching, substrate temperature, geometry, morphology, nanostructured size, laser fluence ordering, reduction time, film thickness, and adhesion. Limitations can be visualized by band misalignment risk, Interfacial defects, internal structural defects, difficult uniformity, high cost, low scalability, over-reduction that increases recombination and limited control over internal stress and defects. It is important to note that the photocurrent densities reported in the literature are measured under different experimental conditions, including variations in the light source, electrolyte composition, applied polarization, and scale of the reference electrode. Therefore, the values in each publication can be interpreted as indicative performance parameters reported by the respective studies, and not as directly comparable benchmarks.

The investigated materials exhibit enhanced structural and electronic characteristics resulting from precise control over film thickness, crystallinity, and surface morphology enabled by these manufacturing methods [87,88,89]. These improvements are reflected in higher photocurrent densities, more efficient charge carrier separation, and improved operational stability under lighting conditions [90].

**Figure 1:**
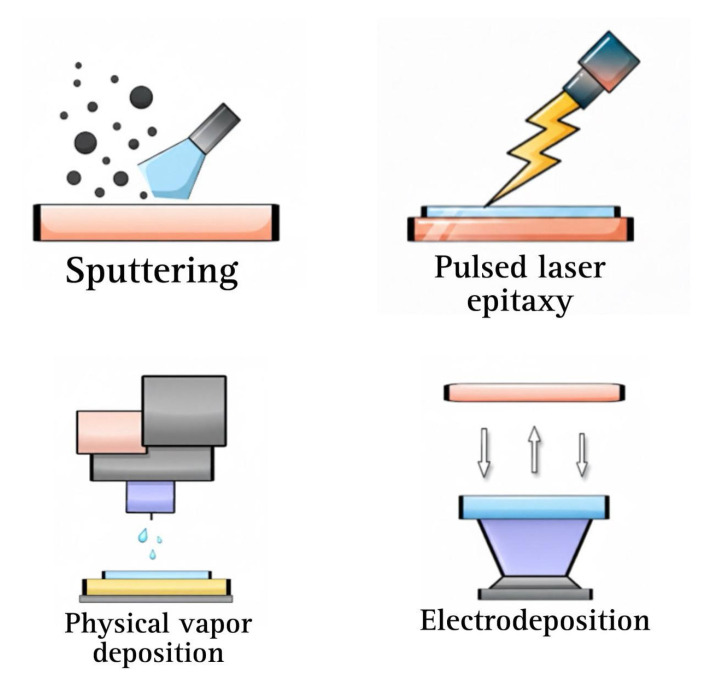
Physical deposition techniques for the fabrication of photoelectrodes used in PEC.

Figure 1 illustrates that physical manufacturing techniques are especially well-suited for the development and optimization of photoelectrochemical electrodes because they provide high material purity, high reproducibility, and compatibility with scalable manufacturing processes.

### 2.2. Chemical Synthesis Methods

The chemical synthesis routes were examined to evaluate their ability to adjust the composition of the crystalline phases and surface chemistry at atomic and molecular levels [91,92,93]. The analysis focuses on techniques such as hydrothermal and solvothermal synthesis, CVD, SILAR, and microemulsion-based methods [94,95,96].

The experimental articles reviewed in this work generally included the preparation of precursor solutions, control of pH and reaction temperature (80–250 °C), and subsequent thermal treatments to achieve the desired crystalline phases. Additional parameters, such as dopant, solvent concentration, polarity, and annealing time, were systematically evaluated due to their influence on charge-carrier lifetime and optical absorption properties [97,98].

Representative chemical synthesis techniques can be enlisted considering controlled chemical doping, colloidal microemulsion, controlled chemical doping, direct solution synthesis, in situ synthesis/electrodeposition, and hydrothermal/solvothermal processing routes. Representative materials in this aspect correspond to plasmonic metals, nanostructures, ferroelectric materials, organic heterojunctions, and organic and inorganic semiconductors [99,100,101,102,103,104]. Along with material composition, and related gains in PEC performance, the bias-free PEC photoelectrode, composite PEC, bandgap engineering, hierarchical PEC structures and PEC photoanodes can be highlighted. The key fabrication parameters can be considered as the grain growth, defect passivation, particle size, porosity, dopant type, concentration, composition, band alignment, mass transfer and crystallite size. The advantages of these advanced materials are related to the photocurrent, enhanced visible absorption, reduced recombination, improved quantum efficiency, enhanced charge separation, high crystallinity and stability. Limitations that should be mentioned are a difficult particle size control, limited visible absorption, interface defects, non-uniform surface distribution and long reaction. Photocurrent values may correspond to various experimental conditions in representative examples of chemical fabrication used in PEC systems from the literature.

**Figure 2:**
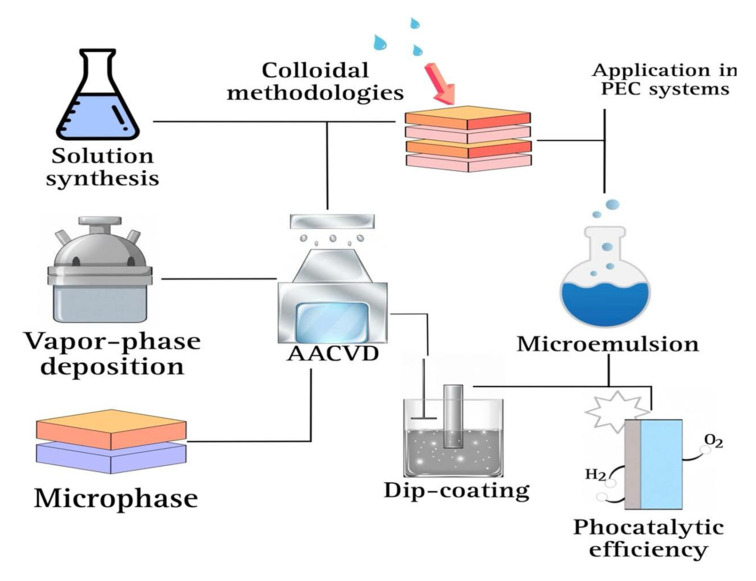
Schematic representation of chemical synthesis and deposition routes, including solution-based methods, colloidal methodologies, and microemulsion techniques, AACVD, and dip-coating, employed in the fabrication of photoelectrodes for photoelectrochemical.

Figure 2 summarizes the studies that employ the chemical synthesis techniques mentioned above in photoelectrochemical systems. These approaches allow precise control of the composition, dopant concentration, and morphology of the nanostructures, resulting in improved charge separation, band gap tuning, and photocurrent response.

### 2.3. Hybrid Manufacturing Approaches

Hybrid manufacturing strategies combine physical and chemical techniques within a unified synthesis framework in order to leverage complementary advantages [105]. The approaches considered in this work include hydrothermal growth coupled with photodeposition [106], SILAR-assisted annealing, immersion electrodeposition, and hybrid processes incorporating chemical reduction and anodization [107].

These techniques were assessed based on their capacity to offer multiscale control over co-catalyst integration, band alignment, and structure, all of which are crucial for reducing recombination losses and enhancing charge transfer [108]. Recent studies have also shown that internal electric fields generated by ferroelectric materials can further promote carrier separation in PEC systems [109]. The analysis examined the relationship between the resulting material architectures, such as doped nanocomposites, cascade bonds, and core–shell heterostructures, and the efficiency of solar-to-hydrogen (STH) conversion [110,111].

Representative hybrid synthesis techniques correspond to controlled chemical doping, SILAR/ligand-assisted deposition, chemical reduction added to thermal assembly, anodization added to chemical and thermal assembly, protective LDH deposition, co-precipitation added to annealing, co-precipitation added to thermal treatment, hydrothermal added to photodeposition, surface deposition, chemical reduction added to hybrid assembly and SILAR added to annealing [112,113,114,115,116,117,118,119,120,121,122]. The elements employed in the fabrication process are plasmonic of monometals and multimetals, multilayered nanostructures, ferroelectric materials integrated in semiconductors, heterojunctions and nanohybrids.

Advantages of hybrid manufacturing approaches can be considered, taking into account bias-free PEC photoelectrode, hybrid photocatalyst, 2D hybrid interfaces, p-n junction PEC photoelectrode, long-term stability, hierarchical porous structures, composite hierarchical electrodes, Type II heterojunction, protective PEC layer, 2D heterostructure PEC, core–shell structures. Key parameters are grain growth, defect passivation, quantum dot size, surface density, charge mobility, dopant distribution field gradient, LDH composition, grain size uniformity, layer homogeneity, nanoparticles shape, band alignment, coating thickness, layer contact quality and base sensitizer interface. Limitations are included in excessive oxide defects, surface defect accumulation, stacking aggregation, instability in alkaline media, LDH dissolution, phase coexistence, photocorrosion, weak interface adhesion, peeling under long operation, band mismatch and sensitizer degradation.

**Figure 3:**
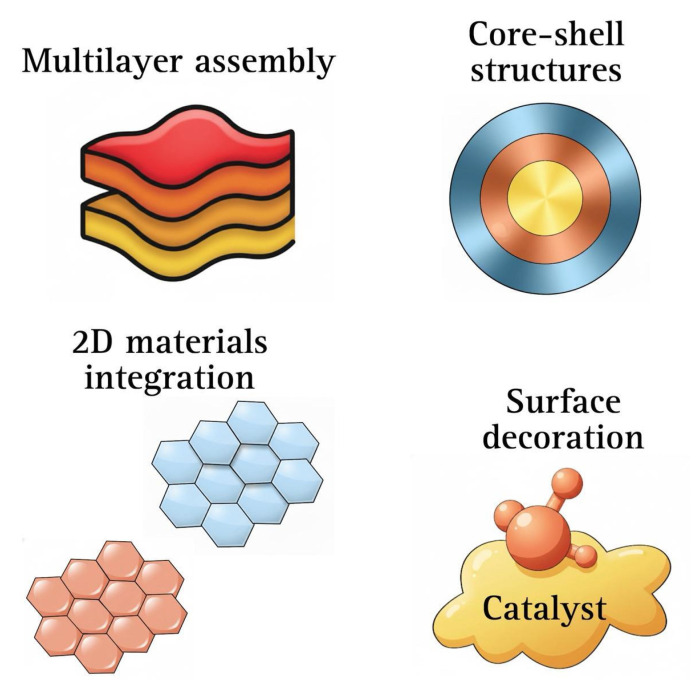
Schematic representation of hybrid physical-chemical strategies used to engineer advanced photoelectrode architectures, including multilayer assemblies, core–shell structures, surface catalyst decoration, and integration of two-dimensional materials.

Figure 3 depicts hybrid fabrication techniques, revealing some underlying synergistic mechanisms that account for their enhanced performance in PEC systems, going beyond the individual examples listed above. These methods typically combine the compositional flexibility and interfacial tunability of chemical synthesis routes with the structural precision of physical deposition techniques. Therefore, band alignment, defect passivation, interfacial charge transfer, and catalytic surface activity can all be simultaneously controlled using hybrid techniques.

Three primary integration principles can be recognized from a design standpoint. First, interface engineering, which optimizes charge separation and surface reaction kinetics by integrating chemically deposited catalysts or secondary semiconductors onto physically deposited photo-absorbers. Second, hierarchical structural engineering, which combines controlled thin-film deposition with chemically generated nanostructured architectures to improve the active surface area, charge transport pathways, and light absorption. Third, functional coupling, which simultaneously enhances stability, catalytic activity, and optical response by incorporating protective layers, co-catalysts, or plasmonic components [123].

These cooperative approaches imply that hybrid fabrication should be understood as a multifunctional materials design paradigm that incorporates structural control, chemical tunability, and interfacial optimization within a single synthesis framework rather than just as a collection of methods. To maximize photoelectrochemical efficiency and stability, such an approach offers a conceptual pathway for designing next-generation photoelectrodes in which morphology, electronic structure, and catalytic functionality are simultaneously engineered [124].

### 2.4. Theoretical and Computational Methods

Different materials that, alongside experimental approaches, theoretical and computational methods, have been used to interpret and predict the physicochemical behavior of photoelectrochemical phenomena [125,126]. The analysis included studies based on DFT, MD, CFD, and optical ray-tracing simulations [127,128].

Some of the parameters evaluated using these techniques included the electronic band structure, charge-carrier transport, interfacial potential distribution, and optical absorption [129]. Furthermore, as complementary tools for material identification and process optimization, machine learning and data-based optimization approaches were investigated [130].

Representative theoretical and computational studies applied to PEC systems include the computational methods employed, target materials, and key predicted outcomes. The studies presented are representative examples selected from the literature to illustrate the different modeling approaches used in PEC research, like ML, DFT, ray tracing, electric field modeling, MD, computational modeling, photoelectronic modeling, CFD and electrostatic modeling. In this respect, PEC applications should consider thermal stability, geometrical optimization, prediction of ideal morphologies, optical modeling, internal field distribution, structural evolution, material ranking, self-healing mechanisms, flow and bubble behavior, electronic structure, dopants, defect energetics, stability, morphology prediction, mass, heat transfer, gas bubble, comparative performance and p-n junction design [45,121,131,132,133,134,135,136,137,138,139,140,141,142,143,144]. The parameters to analyze correspond to phonon modes, lattice distortion, reflection geometry, efficiency dopants, light path optimization, charge separation recombination, surface reconstruction, efficiency factors, redox pathways, gas evolution, turbulence, bandgap, DOS, mobility, defect formation energy, pH dopants, voltage, ohmic losses, flow, heat, stability and potential gradient. Key results may be attributed to reconstruction of the surface under heat, better photon distribution, accurate prediction, angle, reflection, scattering, reduced interfacial loss, temperature-induced changes, clear performance metrics, prediction of self-pair functions, optimized reactor geometry, prediction of increased absorption, identification of stable dopants, rapid screening of materials, higher STH efficiency, optimized PEC operation, identification of global PEC trends, optimized internal fields. Additionally, these approaches develop synthetic data generation, integrate roughness models, full drift diffusion simulation, multi-scale MD, weighted meta-analysis, time-dependent simulations, coupled CFD-DFT strategies, hybrid functionals together with experimental validation, larger supercells, feature engineering, adaptive meshing, multi-physics integration, statistical normalization and coupling with DFT alignment.

Disadvantages are MD time scale limitations, not including charge transport, requiring larger training sets, higher solar capture, oversimplification, limited by small system sizes, variability in reports, no dynamic degradation, CFD ignores nanoscale, DFT approximations understate, limited simulations, model bias, mesh instability, chemical modeling, datasheet heterogeneity and molecular insight.

**Figure 4:**
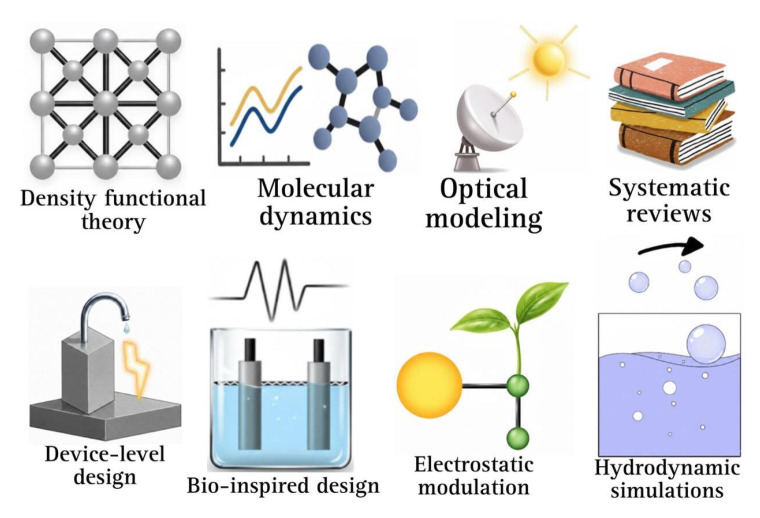
Overview of the theoretical and computational methods used in this work, including DFT, MD, optical modeling, systematic reviews, device-level design, bioinspired design, electrostatic modulation, and hydrodynamic.

Figure 4 illustrates the material systems analyzed, their intended uses, associated limitations, and the resolution of the models employed. This analysis brings together research that uses both theoretical and computational approaches. The models presented offer predictive capabilities for material behavior and device architecture.

In addition to their predictive function, theoretical frameworks such as DFT, MD and CFD are increasingly important for shaping experimental fabrication techniques for photoelectrochemical systems. DFT calculations allow us to predict electronic structures, band alignment, defect formation energies, and adsorption energies of reaction intermediates. This tool helps researchers find promising semiconductor compositions, dopants, and heterostructure configurations before fabricating them in the laboratory. MD simulations also allow us to understand the stability of interfaces and surface interactions when the reactor is operating, while CFD models allow us to improve reactor design and how mass moves through it. All these techniques enable researchers to design photoelectrodes with better charge separation, catalytic activity, and long-term stability more quickly by combining theoretical predictions with experimental fabrication methods [145].

A relevant example of the interaction between theoretical modeling and experimental fabrication can be seen in functional studies, where DFT has been used to design semiconductor heterostructures for photoelectrode cutting PEC applications. In some reports, DFT calculations have been employed to predict favorable band alignment and charge transfer pathways between semiconductor components before synthesis. Examples include theoretical predictions of band shifts and interface energetics that have guided the fabrication of heterostructure photoelectrodes designed to improve charge separation and suppress recombination losses. Based on these results, experimental fabrication strategies such as hybrid deposition and chemical growth methods have been used to construct optimized heterojunction architectures, leading to improved photocurrent density and overall PEC performance [146].

## 3. Comparative Analysis for Fabrication Approaches

As a result of the global trend toward low-carbon energy systems, PEC technologies have become a viable alternative for the direct conversion of solar energy into combustible chemicals [147], particularly hydrogen from water decomposition [148]. However, for a successful implementation, it is necessary to design PEC systems that combine high performance, long-term stability, and costs suitable for practical applications [46,149,150].

Global energy demand continues to rise, and reliance on environmentally damaging fossil fuels persists [151,152]. Consequently, interest in PEC has grown as a potential method for generating low-carbon hydrogen [153]. The direct application of solar energy to drive water-splitting reactions is the driving force behind this interest in technology, which depends on the development of semiconductor interfaces [154] and architectures that enable efficient operation [155]. However, competitive performance can be only achieved through coordinated advances in materials design, interface engineering, and theoretical modeling, which together allow for the optimization of catalytic activity, photoresponse, and long-term stability [156,157].

A major challenge in PEC systems is the development of photoactive materials that optimize light absorption [158] without compromising effective charge separation or increasing recombination losses [90,159]. Instead of relying solely on one methodology, it is necessary to integrate physical, chemical, hybrid, and theoretical approaches whose complementary capabilities allow overcoming the limitations of each [160,161].

Since the production of high-purity thin films with precisely defined structural characteristics and controlled defect densities is highly demanded, physical manufacturing processes remain crucial, such as PLD, which has been particularly effective for the epitaxial growth of photoelectrodes of metal oxides [162]. Some examples are some advanced materials [163,164], as with them it is possible to achieve precise control of stoichiometry, crystallinity, and grain orientation, resulting in films with less interfacial recombination and more efficient charge transport [165,166,167,168].

Another physical fabrication technique is magnetron sputtering, which is widely used [169], offering good film uniformity and easy scalability to larger surface deposits, an essential point for the production of practical photoelectrochemical modules [170,171]. Processing variables such as substrate temperature [172], laser fluence [173], and ambient pressure affect the density, porosity [174], and microstructure of the films [175]. An important aspect is the absorption and catalytic activity of the films [176], which are affected by these variables. As observed in nanostructured systems [177], this technique provides greater band bending and better charge separation through precise control of thickness, doping, and surface nanostructuring [178]. However, physical approaches often have limited flexibility when dealing with complex multicomponent systems or materials requiring spatially gradient compositions [179]. Furthermore, their ability to create local modular chemical environments [180] or to incorporate catalytic nanoclusters directly onto the semiconductor surface or interface is limited, leading to new strategies such as chemical fabrication.

Chemical synthesis methods offer a high degree of control over composition [181], porosity, surface functionalization, and defect chemistry. Hydrothermal and solvothermal techniques, for example, are commonly used to generate high surface area nanostructures such as nanorods, nanosheets, and microspheres, which have been reported to enhance light scattering, charge transport, and catalytic activity in photo-electrochemical systems [181,182,183].

CVD is widely used for the fabrication of layered and two-dimensional photoelectrodes, enabling the integration of carbon-based materials, transition metal dichalcogenides (TMDs), perovskites, and hybrid semiconductors with favorable optoelectronic properties. In parallel, wet-chemical approaches allow controlled incorporation of dopants such as Fe, Co, Mo, W, and selected rare-earth elements to tune band-edge positions, expand visible-light absorption, and enhance oxygen evolution reaction (OER) kinetics. In addition, many chemical routes are compatible with low-temperature, solution-based, and roll-to-roll manufacturing processes, supporting the scalable fabrication of PEC electrodes and facilitating translation toward industrial implementation [184,185,186].

The combination of physical and chemical fabrication methods has become an effective strategy for the design of PEC systems. By combining the structure control of physical deposition techniques with the compositional flexibility and interfacial tuning of chemical synthesis, hybrid approaches enable the design of advanced photoelectrode architectures, including Z-scheme and S-scheme heterojunctions, as well as binary and ternary nanocomposite architectures that enhance charge separation and catalytic activity, which facilitate directional charge transfer and suppress recombination losses by preserving strong redox potential. Also, shell nanostructures provide surface stabilization and increased interfacial trifacial contact between photo absorbers and catalytic sites. Moreover, it is worth mentioning that plasmon-enhanced photoelectrodes based on mono- and multi-metal nanoparticles, where Localized Surface Plasmonic Resonances can enhance optical absorption and promote the generation of hot electrons [138]. Furthermore, co-catalyst-modified interfaces (e.g., NiFeO_x_, Co-LDH, Pt, Pd), which lower reaction overpotentials and enhance oxygen and hydrogen evolution kinetics (OER/HER) [101,187,188].

Hybrid approaches, which combine techniques like light trapping, interfacial engineering, and surface passivation, enable the simultaneous control of morphology, electronic characteristics, and chemical stability, thereby mitigating several factors that limit the performance of PEC systems [189]. As a result, photoelectrodes created with hybrid approaches typically show superior overall PEC performance compared to systems produced using a single fabrication method [190].

In the study and design of PEC materials, machine learning, especially through artificial neural networks, has become more and more important in recent years, supporting conventional experimental methods [191]. Data-driven methods allow for the effective exploration of large search spaces, the identification of ideal synthesis conditions, and the establishment of connections between complex material descriptors and experimental PEC performance [192].

Additionally, reactor geometry, gas bubble dynamics, optical losses, and mass transport processes in PEC cells can be assessed and optimized through the use of optical, mechanical, and fluid dynamics simulations. When combined, computational tools allow for more practical and predictive design of PEC systems, fostering experimentation. From this perspective, an integrated framework is established for the creation and improvement of electrochemical photomaterials through the convergence of physical, chemical, hybrid, and theoretical approaches. This is because, given that a single methodology cannot satisfy the performance requirements related to the PEC of water, progress in this field requires the convergence of atomic-level control, compositional flexibility, scalability, interfacial, and catalytic optimization [193]. At the atomic level, physical deposition techniques allow for precise control of crystallinity, thickness, and defect density. Compositional flexibility and scalability, provided by chemical synthesis routes, enable the evaluation of complex materials and processes across large areas. Interfacial and catalytic optimization, achieved through hybrid engineering strategies, facilitates efficient charge transfer and surface reaction kinetics [172,194,195,196].

Addressing current challenges related to the scalability, long-term stability, and efficiency of PEC systems has required a multidisciplinary approach, as the technology aims to meet the requirements for commercially viable solar hydrogen production, incorporating the reported advances in quantifiable device performance improvements derived from heterojunction research and morphological control [197].

The ongoing advancement of these methodological processes, such as the development of more robust, scalable, and efficient photoelectrochemical systems, together with these advances, increases the potential of PEC technologies for generating solar hydrogen [198]. From an industrial perspective, scalability encompasses not only the ability to create photoelectrodes on remarkable surfaces, but also the ability to maintain reliable performance, robustness, and affordability during mass production. Because they enable the continuous production of thin-film photoelectrodes, techniques such as roll-to-roll processing methods are attractive [199].

We can say that each fabrication strategy offers characteristic advantages, and researchers must consider the trade-offs when selecting an appropriate approach for PEC photoelectrode fabrication. Physical deposition methods typically provide good control over film thickness, crystallinity, and defect density, which favors the achievement of high-quality semiconductor interfaces. However, these techniques involve higher equipment costs and limited composition flexibility. Conversely, solution-based chemical synthesis methods are usually more scalable and cost-effective, allowing for easier tuning of the nanostructures’ composition and morphology, although they do have some disadvantages, offering less precise control over defects and structural uniformity [200]. Hybrid fabrication strategies attempt to balance these considerations by combining the structural precision of physical methods with the compositional versatility and scalability of chemical approaches. Understanding these characteristics is important for selecting the fabrication method that best aligns with the desired performance, scalability, and practical implementation of PEC systems.

Therefore, hybrid process-based design of multicomponent heterostructures, the incorporation of protective and catalytic interlayers, and the development of hierarchical nanostructures capable of enhancing both charge separation and reaction kinetics are expected to be key directions for future research. In addition to the conventional semiconductor systems discussed in this review, recent studies have begun applying these fabrication strategies to emerging classes of materials for photoelectrochemical applications. In particular, single-atom catalysts have garnered significant interest due to their ability to maximize the use of noble metals, and various hybrid fabrication and deposition methods have been explored to anchor isolated active sites on semiconductor photoelectrodes [201]. Similarly, two-dimensional (2D) van der Waals heterostructures have been investigated to improve charge separation and interfacial charge transfer through well-defined layer architectures [202]. Metal–organic frameworks (MOFs) and MOF-derived materials have also emerged as promising platforms due to advantages such as tunable porosity and compositional versatility, which conventional semiconductors lack when using chemical or hybrid manufacturing approaches [203]. These emerging material systems demonstrate how the manufacturing strategies discussed in this review can be extended beyond traditional photoelectrode materials to support the development of next-generation PEC architectures.

## 4. Conclusions

Materials that balance high performance, long-term stability, and appropriate costs for their intended use are crucial for PEC systems to efficiently convert solar energy into hydrogen. To meet these parameters, an integrated and methodical approach to materials design is needed, combining physical, chemical, hybrid, and theoretical methodologies.

Physical fabrication methods are essential for producing high-purity materials with strict structural control. These methods allow for the production of thin films with defined interfaces, excellent crystalline quality, and nanoscale control using techniques such as molecular beam epitaxy, sputtering, and PLD. These characteristics are crucial for increasing the average lifetime of charge carriers, decreasing interfacial recombination, and improving effective charge separation. However, the preference for compositional flexibility in purely physical approaches limits their applicability in complex systems, necessitating their combination with complementary chemical methods, especially in the search for heterostructures or doped materials.

Chemical synthesis methods offer greater flexibility and scalability in composition than purely chemical approaches, by allowing precise control of dopant concentration, surface area, porosity, and band edge alignment through processes such as hydrothermal synthesis, sol–gel, and CVD. Consequently, these capabilities enable the creation of nanostructured structures with quantum confinement that improve light absorption, and chemical processes offer a viable way to produce photoelectrodes in large quantities, essential for the industrial application of PEC technologies. Synthesis, sol–gel, and CVD. Consequently, these capabilities enable the creation of nanostructured structures with quantum confinement that improve light absorption, and chemical processes offer a viable way to produce photoelectrodes in large quantities, essential for the industrial application of PEC technologies.

The mechanistic understanding of PEC systems relies heavily on theoretical and computational approaches. DFT can be useful to explore how dopants, defects, and surface states modify the electronic structure, while MD simulations offer additional insights into the mechanical and thermal stability of materials. The evaluation of light absorption and scattering at the device and reactor levels, as well as the optimization of electrode design and operating conditions, is made possible by optical modeling and CFD. Simultaneously, methods based on machine learning and high-throughput screening are being developed, facilitating experimentation and guiding the logical design of materials by establishing links between structural descriptors and performance metrics.

The practical application of PEC systems is still limited by a number of issues, despite the notable advancements made in recent years. Long-term material stability under operating conditions, effective charge separation across intricate interfaces, and the scalable production of photoelectrodes with regulated morphology and defect density are some of the most important concerns. Improvements in individual materials as well as developments in the integration of fabrication techniques can concurrently optimize structural, electronic, and catalytic properties to meet these challenges.

In this regard, hybrid fabrication techniques are expected to gain greater relevance in photoelectrochemistry research in the future. While PEC systems have shown significant progress, one of the main obstacles to their widespread use is long-term stability. For example, under prolonged illumination and electrochemical operation, semiconductor photoelectrodes are often vulnerable to photo-corrosion which can deteriorate their structural and electrical characteristics. Furthermore, surface reconstruction processes can occur during operation, modifying active sites and affecting catalytic performance. The potential separation or degradation of cocatalyst layers deposited on the photoelectrode’s surface is another important factor, especially in cases of weak interfacial bonds. In order to overcome these limitations, several recent studies have explored various strategies, including the deposition of protective oxide layers, the optimization of improved interfaces, and the design of more stable cocatalyst integration approaches. These strategies aim to improve the durability of PEC photoelectrodes while maintaining high catalytic activity and efficient charge transfer.

Hybrid strategies enable the simultaneous engineering of light absorption, charge transport, catalytic activity, and corrosion resistance in multifunctional photoelectrode architectures by combining physical deposition techniques with chemical synthesis approaches. Therefore, the logical design of multicomponent heterostructures, the addition of protective and catalytic interlayers, and the creation of hierarchical nanostructures that can enhance both charge separation and reaction kinetics may be the main areas of future research.

Combining data-driven methods and computational modeling with experimental fabrication techniques is another promising avenue. The identification of ideal material combinations and fabrication parameters can be expedited by machine learning, multiscale simulations, and predictive modeling, allowing for more effective design of hybrid PEC systems. Thus, a route toward scalable and highly effective photoelectrochemical technologies for sustainable hydrogen production may be made possible by the convergence of sophisticated fabrication techniques, interface engineering, and computational optimization.

## Figures and Tables

**Table 1:** Representative materials used in PEC water splitting systems.

Material	Type of Photoelectrode	Key Properties	Typical Role in PEC
TiO_2_	n-type semiconductor	High stability, wide bandgap	Photoanode
WO_3_	n-type semiconductor	Good electron mobility, visible absorption	Photoanode
BiVO_4_	n-type semiconductor	Visible-light absorption, good OER activity	Photoanode
Fe_2_O_3_	n-type semiconductor	Abundant, narrow bandgap	Photoanode
Cu_2_O	p-type semiconductor	Suitable bandgap, high absorption	Photoanode
ZnO	n-type semiconductor	High electron mobility	Photoanode
C_2_N_4_	Polymeric semiconductor	Visible-light response	Hybrid heterostructures
Perovskite oxides	Mixed systems	Tunable band structure	Advanced PEC systems
Plasmonic nanoparticles	Metals	Tunable optical response	Advanced PEC system

## Data Availability

No new data were created or analyzed in this study. Data sharing is not applicable to this article.
